# Mycotoxin Biotransformation by Native and Commercial Enzymes: Present and Future Perspectives

**DOI:** 10.3390/toxins9040111

**Published:** 2017-03-24

**Authors:** Martina Loi, Francesca Fanelli, Vania C. Liuzzi, Antonio F. Logrieco, Giuseppina Mulè

**Affiliations:** 1 Institute of Sciences of Food Production, National Research Council, via Amendola 122/O, Bari 70126, Italy; martina.loi@ispa.cnr.it (M.L.); vania.liuzzi@ispa.cnr.it (V.C.L.); antonio.logrieco@ispa.cnr.it (A.F.L.); giuseppina.mule@ispa.cnr.it (G.M.); 2 Department of Economics, University of Foggia, via Napoli 25, Foggia 71122, Italy

**Keywords:** mycotoxins, biotransformation, degradation, enzymes, application

## Abstract

Worldwide mycotoxins contamination has a significant impact on animal and human health, and leads to economic losses accounted for billions of dollars annually. Since the application of pre- and post- harvest strategies, including chemical or physical removal, are not sufficiently effective, biological transformation is considered the most promising yet challenging approach to reduce mycotoxins accumulation. Although several microorganisms were reported to degrade mycotoxins, only a few enzymes have been identified, purified and characterized for this activity. This review focuses on the biotransformation of mycotoxins performed with purified enzymes isolated from bacteria, fungi and plants, whose activity was validated in in vitro and in vivo assays, including patented ones and commercial preparations. Furthermore, we will present some applications for detoxifying enzymes in food, feed, biogas and biofuel industries, describing their limitation and potentialities.

## 1. Introduction

Mycotoxins are secondary toxic metabolites produced by filamentous fungi mainly belonging to *Fusarium*, *Aspergillus* and *Penicillium* genera. They infect cereals, seeds and fruits both in the field and during storage, and can be found as common contaminants in food and feed supply chains [1,2].

Mycotoxins poisoning of staple food commodities has a significant impact on worldwide health, especially in developing countries [3,4]. Both animals and humans may develop acute and chronic mycotoxicosis, depending on several factors, which include the type of mycotoxin, the amount and the duration of the exposure, etc. Mycotoxins can be classified as hepatotoxins, nephrotoxins, neurotoxins, immunotoxins and so forth, based on the organ they affect [1]. Main target tissues include gastrointestinal and breathing apparatus, endocrine, exocrine, reproductive, nervous and immune system [5,6]. Aflatoxin B_1_ (AFB_1_) is the most toxic mycotoxin and has been classified in group 1, carcinogenic to humans, by the International Agency of Research on Cancer (IARC) in 2002 [7]. Other mycotoxins, such as aflatoxin M_1_ (AFM_1_), ochratoxin A (OTA) and fumonisin B_1_ (FB_1_), are classified within group 2B, thus possibly carcinogenic to humans, due to the limited availability of their toxicological data [7,8,9].

Synergistic and additive effects of different mycotoxins have been documented; however, the worsening and toxic outcome of multiple exposure cannot be predicted by summing up the individual toxicities. Furthermore, the mechanisms of interactions among toxins are still poorly understood [10]. In rats FB_1_ synergistically promotes liver lesions, hepatocyte dysplasia and, by long term exposure, tumors initiated by AFB_1_ [11]. AFB_1_ also acts synergistically with zearalenone (ZEN) in decreasing egg production, feed intake, feed conversion ratio and eggshell strength in birds [12]. In vitro cytotoxicity of AFM_1_ on Caco-2 cells is greatly enhanced by the presence of OTA, ZEN and/or α-zearalenol (α-ZEL), which often co-contaminate milk and infant formulas [13]. OTA and citrinin have often been reported to act in a synergistic mode in relation to their cytotoxic [14,15,16,17] and genotoxic effects [18].

Global trade significantly contributes to mycotoxin spread. The economic losses associated with mycotoxin contamination in commodities account for billions of dollars annually [19]. They can be categorized into direct and indirect losses: direct losses are related to lowered crop yields, reduction of animal performance and costs derived from diseases for livestock producers; indirect losses are very challenging to quantify, and are linked to the increased use of fungicide, the reduction of the marketable value of the commodities, the management, health-care, veterinary-care costs, and investments in the development of reducing strategies and in research programs.

Mycotoxins levels are regulated in many countries worldwide. The European Union has implemented the most extensive and detailed food regulation for mycotoxins within the Commission Regulation (EC) No. 1881/2006 [20], which set mycotoxins maximum levels discriminating among food products and consumers (e.g., adults or infants). Indicative levels in cereals and cereals products [21] have been established for the trichothecenes T-2 and HT-2 toxins (T-2, HT-2). A scientific opinion on their toxicity was released in 2001 by the European Commission Scientific Committee on Food (SCF) [22], highlighting the need for further studies on the occurrence, daily intake, analytical methods development and induced hematotoxicity and immunotoxicity. However, for other so-called emerging mycotoxins (e.g., enniatins, beauvericin, fusaric acid and moniliformin), whose increasing occurrence has been clearly evidenced, maximum levels have not been yet established. This delay exists since certified analytical methods for their determination, complete surveys on their occurrence and defined scientific opinions on their toxic effect and health associated risks are still being developed [21,23].

Mycotoxin contamination can be prevented in the field through the application of good agricultural practices (GAPs), such as the choice of resistant varieties, harvesting at the right time, crop rotation, and the use of fungicides [20,24]. Nevertheless, pre-harvest strategies are not completely effective, and fungal contamination of raw materials can lead to mycotoxin accumulation during storage. Furthermore, mycotoxins are extremely stable and resistant to the commonly used physical and chemical treatments of food and feed processing.

Since the application of GAPs, proper storage and risk management procedures might only mitigate mycotoxin occurrence, the development of alternative strategies to reduce mycotoxins contamination is considered a relevant, innovative, urgent, yet challenging research topic.

In this review, we will briefly present the current methods to reduce mycotoxins contaminations in food and feed, and then we will focus on the biotransformation of mycotoxins performed with purified enzymes isolated from bacteria, fungi and plants, whose activity was validated in in vitro and in vivo assays, including patented ones and commercial preparations. We will also describe enzyme potential applications and limitations in food, feed and bioenergy in compliance with the European Regulation.

### Methods for Mycotoxins Reduction

Current methods to reduce mycotoxin contamination in food and feed can be classified into physical, chemical and biological. It must be underlined that EU regulation does not authorize any detoxification methods for those commodities, intended for food production, exceeding mycotoxins limit levels [20].

(1) Physical methods comprise the mechanical removal of highly contaminated fractions from raw materials (by sorting, cleaning, milling, dehulling), the application of heat, the irradiation and the use of adsorbents, which is still limited to feed production [25]. The latter approach is considered promising, although some possible negative drawbacks related to unspecific binding of essential nutrients and antibiotics exist. In addition, these adsorbents have diversified efficacy towards different classes of mycotoxins, with trichothecenes being the most difficult to target. Furthermore, since co-occurrence is much more common than individual contamination, complementary reduction strategies should be implemented.

Recently, the application of unconventional strategies such as cold plasma [26,27,28], photoirradiation [29,30] and microwave treatments [31] have been proposed and/or introduced in food processing, as sterilization or degrading methods. Nevertheless, the partial knowledge about degradation products (DPs) and the nutritional/organoleptic changes induced by these treatments still confine their application.

(2) Chemical methods such as ammoniation [32], acid treatments [33], alkaline hydrolysis [34], peroxidation [35], ozonation [36], and the use of bisulphites [37] have been tested, but their application in food and feed is limited due to their potential toxicity, poor efficacy, high costs and negative effects on the quality of raw materials.

(3) Biological methods consist of the use of microorganisms or enzymes, which are able to metabolize, destroy or deactivate toxins into stable, less toxic, up to harmless compounds [38]. Biological agents and their enzymes allow a specific, most likely irreversible, environmental friendly and effective approach, with minor impact on food sensory and nutritional quality with respect to chemical ones.

## 2. Mycotoxin Biotransformation by Enzymes

Mycotoxin biotransformation is defined as “the degradation of mycotoxins into non-toxic metabolites by using bacteria/fungi or enzymes” [39]. The possibility to use living microorganisms as whole cell biocatalysts for mycotoxins degradation has cost advantages. This represents a valid strategy, especially if multi step reactions are required, or if the microorganism is already implemented within industrial processes [40,41]. On the other hand, in case of high levels of mycotoxins contamination, the growth and physiology of such microorganisms might be altered or inhibited, thus requiring longer time for adaptation before achieving satisfactory decontamination levels.

The majority of the research papers which describes microbial degrading activity, rarely discriminates between physical adsorption and enzymatic degradation. This greatly complicates the identification of DPs and the evaluation of their toxicity. This knowledge is relevant in the evaluation of biotrasforming enzymes, especially since not every reaction leads to a real detoxification. Indeed, the metabolized mycotoxin can acquire greater toxic properties than the parent compound. This is the case, for example, of ZEN biotransformation performed in vivo by yeasts, which reduce the toxin to α-ZEL, actually more estrogenic than ZEN [42].

The identification and characterization of a degrading enzyme (DE) can be challenging and time consuming, but it is a necessary step to understand the mechanism of degradation, towards its optimization and the development of mycotoxins reducing methods. Enzymes guarantee reproducible and homogeneous performances, with ease-of-handling, no risks of contamination and no safety concerns for operators compared to the use of living microorganisms.

Screening microbial population from different mycotoxin-contaminated environment is an efficient, fast and promising strategy to discover degrading microbes and activities [43,44,45], which could be enhanced by coupling advanced approaches, such as metagenomics or functional metagenomics.

In addition, genetic engineering enables to clone and express heterologous enzymes in bacterial, yeast, fungal and plant cells for massive, less expensive and less laborious productions. If no structural-functional data are available, enzyme efficiency, stability and tolerance to organic solvents can also be improved by computational approaches, aiming at targeted or combinatorial semi-rational mutagenesis.

Many enzymes have been reported to remove or reduce mycotoxin contamination both in vitro and in real matrices. Nonetheless, their application in feed is very limited, due to the lack of information about the potential toxic effects of generated products and their influence on nutritional quality of feed. These data are mandatory to be authorized as possible biotransforming agent in Europe [39].

Very few commercial biotransforming feed additives are available: Mycofix^®^, FUMzyme^®^, Biomin^®^ BBSH 797 and Biomin^®^ MTV (Biomin Holding GmbH, Getzersdorf, Austria) are some examples, but only FUMzyme^®^ exploits a purified enzyme, an esterase, to perform fumonisin degradation. Below we will discuss in details the biotransformation of the main mycotoxins by native and commercial enzymes.

### 2.1. Aflatoxins

**Figure 1:**
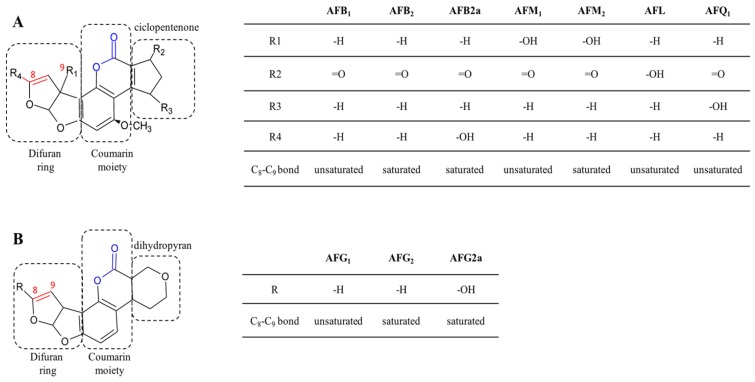
Chemical structure and features of ciclopentenone (**A**) and difurocoumarolactone (**B**) aflatoxin (AF) series. Coloured bonds indicate reactive groups involved in AFs toxicity. The double bond leading to 8,9-epoxide upon metabolic activation is indicated in red, while the lactone bond is indicated in blue. Tables show substituent groups and saturation of the C_8_-C_9_ bond in different AF analogues.

The term aflatoxins (AFs) includes more than 20 fungal secondary metabolites produced by fungi belonging to *Aspergillus* genus [1]. They are classified into two main groups according to their chemical structure. The difurocoumarocyclopentenone group includes AFB_1_, aflatoxin B_2_, aflatoxin B2a, AFM_1_, aflatoxin M_2_, aflatoxin Q_1_ (AFQ_1_), and aflatoxicol (AFL), while the difurocoumarolactone group comprises aflatoxin G_1_, aflatoxin G_2_ and aflatoxin G2a (Figure 1).

While AFs of the B and G series co-occur in cereals and their derived products, fruits, oilseeds, nuts, tobacco and spices, AFM_1_ AFM_2_, AFL and AFQ_1_ are detected in food as carry-over products of AFB_1_ contaminated feeds. In vivo AFB_1_ is readily metabolized through hydroxylation (to AFM_1_ or, to a lesser extent, to AFQ_1_) or reduction (to AFL).

AFs are difuranocoumarin derivatives composed by two furan rings, linked together to a coumarin moiety. Furofuran and cumarin rings are arranged in a planar configuration which is responsible for conjugation leading to the typical AFs fluorescence.

The furofuran ring has been recognized as responsible for the toxic and carcinogenic activity upon metabolic activation of the C_8_-C_9_ double bond to 8–9 epoxide [9] (Figure 1).

The epoxidation is a crucial reaction for AFs carcinogenicity, since it allows the binding to N7-guanine and the subsequent G to T transversions in the DNA molecule [46]. Activated AFs are also able to form schiff bases with cellular and microsomal proteins (via methionine, histidine and lysine), thus leading to acute toxicity [47]. The lactone ring also plays a role in AFs toxicity and carcinogenicity: upon ammoniation it is hydrolyzed, forming aflatoxin D_1_ (AFD_1_) which still retains the 8,9-dihydrofuran double bond; AFD_1_ lacks the strong in vivo DNA binding activity of AFB_1_, demonstrating that DNA alkylation depends upon both difuranocumarin and lactone moieties [48].

Several authors addressed lactone hydrolysis, reduction or addition reactions as possible mechanisms of degradation, since the putative hydrolyzed products showed greatly reduced mutagenic activity in vitro [49,50].

Table 1 summarizes the purified enzymes identified as capable of degrading AFs, and their main features. Although direct comparison is not possible, since the enzymes and the experimental conditions used by the authors are not equivalent, reaction parameters and AFs concentrations are indicated.

Most of these enzymes are comprised in the oxidoreductase (EC 1) group. The first identified was the AF oxidase enzyme (AFO) isolated from the edible fungus *Armillariella tabescens* by Liu and colleagues in 1998 [51]. AFO is an oxygen dependent reductase, releasing H_2_O_2_ as by product [51,60]. One mg/mL of enriched preparation and 30 min of incubation at 28 °C were needed to completely degrade 150 ng of AFB_1_. AFO did not affect AFB_1_ fluorescence properties, indicating that the conjugation system, between the coumarin moiety and the lactone ring, was not disrupted by the degrading reaction. The mechanism proposed by the authors consisted of the enzymatic cleavage of the bisfuran ring. This hypothesis, although not yet experimentally verified, is in agreement with the reduced mutagenicity, toxicity and genotoxicity of the DPs and with their differential pulse voltammetry response similar to furofuran analogs [61,62]. Products of AFO treatment were found to exert neither liver toxicity in rats, nor mutagenicity activity on *Salmonella typhimurium* TA98, and not to be genotoxic to chicken embryos [61].

Three different peroxidases (EC 1.11.1.7) were also reported to possess AFB_1_ degrading capabilities. They were extracted and purified from horseradish (*Armoracia rusticana*) [52], *Phanerochaete sordida* YK-624 [63] and *Pleurotus ostreatus* [56]. The horseradish peroxidase was tested in vitro towards AFB_1_, which was reduced by 42% after 1h with 0.2 U/mL of enzyme. Similar results were also obtained in real matrix: AFB_1_ was reduced by 41% in artificially spiked groundnut [64]. A lowered toxicity of the DPs was also registered by inhibition growth assay on *Bacillus megaterium* [51]. The two manganese peroxidases (MnPs) from the two different white rot fungi were studied [56,63] in in vitro assays with similar reaction conditions, allowing a direct comparison. They were tested towards 0.3 µg/mL of AFB_1_, achieving 86% reduction after 24 h of incubation, and up to 90% after 48 h by using the MnP from *P. sordida YK-624* and the one from *P. ostreatus* respectively. Wang and colleagues [63] also registered 69.2% reduction of mutagenicity of DPs compared to AFB_1_, based on the umu test performed on *Salmonella typhimurium* TA1535 and on S9 liver homogenate. According to Proton Nuclear Magnetic Resonance (^1^H-NMR) and high resolution electrospray ionization mass spectrometry (HR-ESI-MS) analysis, the authors hypothesized that MnP treatment converted AFB_1_ to AFB_1_-8,9-dihydrodiol.

Laccases (EC 1.10.3.2) (LCs) have been intensively used in bioremediation [65] and were recently proposed for ZEN and AFs biotransformation [59,66,67].

Alberts and colleagues [53] made as firsts a decisive step towards the correlation between AFs degradation and LCs activity. The authors used different LCs, including native LCs from representatives of *Peniophora* genus and *P. ostreatus*, one commercial LC from *Trametes versicolor*, and one recombinant LC from *Aspergillus niger*. By treatment with 1 U/mL of the commercial preparation AFB_1_ (1.4 µg/mL) was reduced by 87.34% after 3 days of incubation at 30 °C. DPs were not identified, but proved to be less mutagenic than the parent compound. However, none of the preparations used by the authors was purified to homogeneity. The commercial product from *T. versicolor* was indeed an enriched preparation, which included additional proteins and different laccase isoforms [68]; thus, an unambiguous assignment of the degrading activity to a specific LCs would be improper.

Recently the effectiveness of purified LCs towards AFs and ZEN was acknowledged [58,67,68]. Pure LCs from *P. pulmonarius* and *P. eryngii* were used by Loi and colleagues [58,59] towards both AFB_1_ and AFM_1_. The authors performed in vitro and *in matrix* tests with 1 µg/mL of AFB_1_ and 0.05 µg/mL for AFM_1_. Interestingly, they reported that, while LC alone is poorly able to degrade these toxins, the addition of a redox mediator at 10 mM concentration increased the degrading percentages from 23% up to 90% for AFB_1_, and up to 100% for AFM_1_ after 72 h [58,59].

The laccase-mediator approach patented by Novozymes in 2008 [54], consisted of the use of LCs, preferably from *Streptomyces coelicolor*, and methylsyringate as mediator. In this case, AFB_1_ was completely removed after 24 h of incubation with 0.1 mg/mL of enzyme and 0.2 mM of mediator at 37 °C.

Members of the reductase family have also been studied for AFs degrading capabilities. Among these, the F420H2-dependent reductases (EC 1.5.8.), isolated from *Mycobacterium smegmatis*, were tested towards AFB_1_, AFB_2_, AFG_1_ and AFG_2_; no details were provided in relation to efficacy and assay conditions [55].

Besides oxidases above described, Guan et al. [69] identified the myxobacteria aflatoxin DE (MADE), isolated from *Myxococcus fulvus*, which was further characterized in 2011 [57]. Pure MADE (100 U/mL) was tested towards 0.1 µg/mL each of AFG_1_ and AFM_1_, achieving 98 and 97% of degradation after 48 h of incubation. No further details about the nature of the enzyme, nor about the DPs were given.

### 2.2. Ochratoxin A

**Figure 2:**
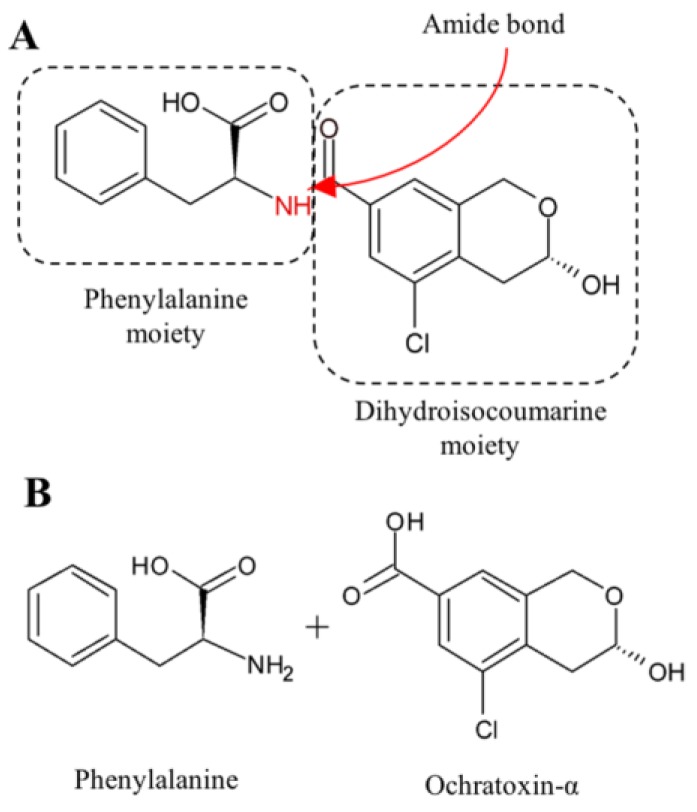
Chemical structures of (**A**) ochratoxin A and (**B**) its degradation products, ochratoxin-α and phenylalanine. The amide bond hydrolyzed by the main degrading pathway is indicated in red.

OTA is a phenylalanine-dihydroisocoumarine derivative, composed of a 7-carboxy-5-chloro-8-hydroxy-3,4-dihydro-3-R-methylisocoumarin (ochratoxin α—OTα) moiety and a l-β-phenylalanine molecule (Phe), which are linked at the 7-carboxy group by an amide bond (Figure 2).

Due to its structural analogy to the amino acid Phe, the toxin can competitively inhibit tRNA phenylalanine synthetases and, consequently, block protein synthesis [70]. Furthermore, OTA causes the formation of DNA adducts, indirect oxidative DNA damage and activates a network of interacting epigenetic mechanisms [71,72].

The main OTA detoxification pathway consists in the hydrolysis of the amide bond between the isocoumarin residue and phenylalanine, resulting in the formation of Phe and OTα (Figure 2). The former is considered to be a non-toxic compound, with a 10-times shorter elimination half-life than OTA [73].

Numerous enzymes were hypothesized to hydrolyze the OTA amide bond, but only few of them were isolated and characterized [74].

Two classes of carboxypeptidases (EC 3.4) have been associated with OTA degradation: carboxypeptidase A (CPA) and carboxypeptidase Y (CPY) class. CPA uses one zinc ion within the protein for hydrolysis (EC 3.4.24), while CPY is a serine-type carboxypeptidase (EC 3.4.16) and does not contain any zinc ion in its active site. The first peptidase reported as able to hydrolyze OTA was a CPA isolated from bovine pancreas, which resulted able to perform the degrading reaction with a Km value of 1.5 × 10^−4^ M at 25 °C [75]. A CPY isolated from *Saccharomyces cerevisiae* was demonstrated to hydrolyze OTA with optimum at pH 5.6 and 37 °C; its specific activity was very low considering that only 52% of OTA was converted into OTα after five days of incubation [74]. The same enzyme was efficiently immobilized on electroactive surfaces in order to develop a biosensor system for the direct detection of OTA in olive oil, with promising results [76]. Many carboxypeptidases have high optimal reaction temperatures (30 °C or higher); this might not hamper detoxifying applications for food and feed [25].

Other enzymes are also able to perform OTA hydrolysis, such as lipases (EC 3.1), amidases (EC 3.5) and several commercial proteases (EC 3.4) [77,78,79].

By screening different commercial hydrolases, a lipase preparation from *Aspergillus niger* (Amano A) was shown to hydrolyze OTA into OTα and Phe. Single-step purification, by anion exchange chromatography, allowed the isolation of the pure protein. The lipase nature of the enzyme was confirmed by assaying the cleavage of p-nitrophenyl palmitate. The purified enzyme resulted able to completely hydrolyze 50 µg of OTA into OTα after 120 min of incubation, in 1 ml of reaction mixture [77].

Several commercial proteases were also reported to hydrolyze OTA to OTα, such as Protease A from *A. niger* and pancreatin from porcine pancreas. These enzymes showed a significant hydrolytic activity at pH 7.5, which resulted in the cleavage of 87.3% and 43.4% of 1 µg of OTA respectively, after 25 h of incubation in 1 ml reaction mixture [78].

Finally, an amidase and the feed or food additive comprising it, capable of degrading OTA, were patented by Dalsgaard et al. in 2010 [79]. The protein, named amidase 2, is encoded by an open reading frame of *A. niger*. The degrading assay was performed in 300 µL of reaction mixture, containing 160 ng/mL amidase 2 and 50 µg/mL of OTA. This enzyme was able to reduce OTA concentration by 83%. Amidase 2 was tested also in some food preparations. The patented additive decreased OTA content, from 47 ppb to undetectable level (<2 ppb), after 2.5 h-incubation in contaminated milk. Similarly, OTA concentration in corn flour was reduced from 38 ppm to less than 2 ppb after 20 h of incubation.

### 2.3. Fumonisins

**Figure 3:**
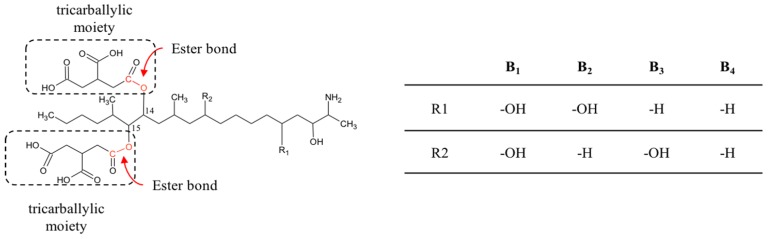
Type B fumonisins chemical structure. The ester bonds hydrolyzed by the main degrading pathways, leading to the formation of HFB_1_ and the two tricarballylic acid moieties, are indicated in red. The table shows substituent groups of different fumonisin analogues.

Fumonisins are a group of mycotoxins associated with several mycotoxicoses, including equine leukoencephalomalacia, porcine pulmonary edema and experimental kidney and liver cancer in rats [80]. Chemically, fumonisins are diesters of propane-1,2,3-tricarboxylic acid and similar long-chain aminopolyol backbones. Structurally they resemble the sphingoid bases sphinganine (SA) and sphingosine (SO), with tricarboxylic acid groups added at the C_14_ and C_15_ positions (Figure 3). This structural similarity is responsible for the fumonisin mechanism of action. It was described to act by disturbing the sphingolipids metabolism, by inhibiting the enzyme ceramide synthase, and leading to accumulation of sphinganine in cells and tissues [81,82,83].

There are at least 28 different forms of fumonisins, designated as A-series, B-series, C-series, and P-series [84]. The B-series (FB) is the most abundant and important with respect to toxicity.

Pre-harvest strategies to reduce fumonisins contamination are based on the bio-control of the spread of fumonisin-producing fungi. Post-harvest methods include the application of natural clay adsorbents during food processing. While they do not lead to a real detoxification of fumonisin [85,86], different microorganisms were reported to transform these class of mycotoxins. Among them, only few enzymes have been identified, molecularly and biochemically characterized, and patented.

FB_1_ was reported to be degraded by the consecutive action of a carboxylesterase (EC 3.1.1) [87] and an aminotransferase (EC 2.6.1) [88,89] (Table 2).

By the deesterification action of the carboxylesterase, the two tricarballylic acid moieties are released, resulting in hydrolyzed FB_1_ (HFB_1_), also known as aminopentol 1 (AP1). After the oxidative deamination, HFB_1_ is converted to *N*-acetyl HFB_1_ and 2-oxo-12,16-dimethyl-3,5,10,14,15-icosanepentol hemiketal [88]. The action of these enzymes, isolated from *Sphingomonas* sp. ATCC 55552 [90], was worldwide patented in 1994 [89].

The same degrading activity was shown by *Sphingopyxis* sp. MTA144, isolated from composted earth [92]. In this strain the two key DEs were identified within a gene cluster: *fumD*, encoding a type B carboxylesterase, and *fumI*, encoding an aminotransferase [80,93,94]. The authors also demonstrated the degrading activities of the two recombinant enzymes by in vitro assays. The first enzyme, encoded by *fumD*, was able to catalyze the complete deesterification of FB_1_ (3.34 µg/mL) to HFB_1_ within 15 min. The oxygen independent aminotransferase, encoded by *fumI*, was shown to deaminate HFB_1_ (8.9 µg/mL), in presence of pyruvate and pyridoxal phosphate, within 44 h. Furthermore, the authors described the additional genes located in this putative degrading cluster identified by genome walking (NCBI Accession n. FJ426269). The cluster includes one transporter, one permease, dehydrogenases, and transcriptional regulators and was U.S. patented by Moll et al. within the “Method for the production of an additive for the enzymatic decomposition of mycotoxins, additive and use thereof” [95].

The esterase (EC 3.1.1.87) from *Sphingopyxis* sp. MTA144, produced by a genetically modified strain of *Komagataella pastoris* (formerly *Pichia pastoris*), has been included in a patented formulation, FUMzyme^®^ (Biomin Holding GmbH, Getzersdorf, Austria). This enzyme-based feed additive, whose safety and efficacy has been recently evaluated by EFSA [91], is intended to degrade fumonisins found as contaminants in feeds for growing pigs. The esterase partially degrades FB_1_ and related fumonisins by cleavage of the diester bonds and release of the tricarballylic acid.

### 2.4. Trichothecenes

**Figure 4:**
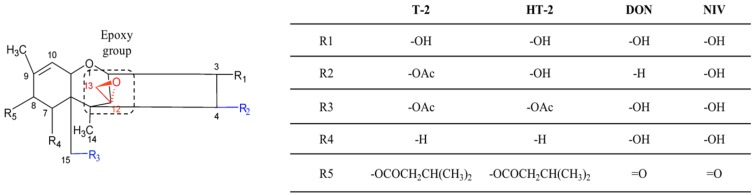
Chemical structure of trichothecenes. Groups responsible for trichothecenes toxicity are highlighted in red (epoxide) and blue (substituent groups, see the text for further details). The table shows substituent groups of different trichothecene analogues.

Trichothecenes are a large group of sesquiterpenoid metabolites sharing a common core structure comprised in a rigid tetracyclic ring (Figure 4). To date, more than 190 trichothecenes and derivatives have been described [96,97].

Their synthesis starts with the formation of trichodiene via the cyclization of farnesyl pyrophosphate. Trichodiene then undergoes a series of oxygenation, cyclizations, isomerization and esterification needed for the bioactivation of the molecule [98].

Trichothecenes are classified into 4 groups according to the functional groups associated to the core molecule: type A trichothecenes (e.g., T-2 and HT-2) do not contain a carbonyl group at the C-8 position; type B trichothecenes (mainly represented by deoxynivalenol (DON) and nivalenol (NIV) contain a carbonyl group at the C-8 position; trichothecenes of type C (e.g., crotocin and baccharin) have an additional epoxy ring between C-7 and C-8, or between C-9 and C-10; trichothecenes of type D (e.g., satratoxin and roridin), contain a macrocyclic ring between C-4 and C-15 (Figure 4).

The results of trichothecenes exposure on eukaryotic cells was reviewed by Rocha et al. [99], while T-2 and HT-2 toxicity data were reported to EFSA in 2010 by Schuhmacher-Wolz and colleagues [100]. Trichothecenes are not degraded during normal food processing, they are stable at neutral and acidic pH and, consequently, they are not hydrolyzed in the stomach after ingestion [99].

Type A trichothecenes arise special interest, being more toxic than the other food-borne trichothecenes [101]. Among the effects exerted by T-2 and HT-2 toxins several studies reported the inhibition of DNA, RNA and protein synthesis, and apoptotic effects in mammalian cell cultures in vitro and ex vivo [22,102].

T-2 is rapidly metabolized to HT-2 in the gut. Data indicate that these toxins induce acute toxic effects with similar severity. For these reasons, the toxicity of T-2 in vivo is considered to include that of HT-2 [102]. Type B trichothecene have a relatively low toxicity compared to type A trichothecene, but it varies depending on the species and the cell type. The general toxic mechanism of trichothecenes is induced by the interference with protein synthesis, through 60S ribosome binding, leading to translation inhibition [103].

The 12,13-epoxy ring is the most important structural determinant of trichothecenes toxicity. In addition, the presence of hydroxyl or acetyl groups at appropriate positions on the trichothecene core [104], the presence of substituents on C_15_ and C_4_ [105,106], and of the side groups, are implicated in defining the degree of toxicity [107]. Both acetylation and de-acetylation may reduce toxicity of trichothecenes, as well as epimerization, oxidation [108,109,110].

Trichothecenes are noticeably stable molecules under different treatments, including both thermal and chemical ones. Several microorganisms, isolated from disparate complex environments, such as rumen or soil, have been reported to degrade trichothecenes into de-acetylated and/or de-epoxidated products [25]. However, the majority of the studies focused on this topic never achieved the identification of the enzymes responsible for the biotransformation. Nevertheless, considering the reaction products, acetylase, deacetylase or de-epoxidase activities are surely involved in the process.

In addition, hydroxylation and glycosylation of trichothecenes generate less toxic derivatives [111]. In this case, these compounds might undergo reverse reaction in the digestive tract of humans and animals, limiting the use of these enzymes at least in feed-related applications.

Recently, the bacterial cytochrome P450 system (EC 1.14) from *Sphingomonas* sp. strain KSM1, was reconstructed in vitro and demonstrated to hydroxylate DON, NIV and 3-acetyl DON [112] (Table 3). The system includes the cytP450, encoded by the gene ddnA, and the endogenous redox partners. The DON catabolic product, 16-HDON, is used by the *Sphingomonas* strain as a carbon source, and was shown to exert a reduced phytotoxicity to wheat.

Poppengberger et al. [113] identified a UDP-glycosyltransferase from *Arabidopsis thaliana* (the deoxynivalenol-glucosyl-transferase DOGT1) able to catalyze the transfer of glucose from UDP-glucose to the hydroxyl group at C3 of DON forming 3-*O*-glucopyranosyl-4-DON. The overexpression of this enzyme in *A. thaliana* enhanced DON resistance. The reaction products were identified by in vitro assays, using the enzyme purified from recombinant *Escherichia coli* cells.

Commercial products containing biotransformant agents have already been developed and patented. As an example, Biomin^®^ BBSH 797 includes a pure culture of *Eubacterium* BBSH 797 isolated from bovine rumen fluid; it is capable of converting DON into DOM-1 and with de-epoxydase activity towards NIV, T-2, tetraol, scirpentriol, and HT-2. Nevertheless, purified enzymes with specific trichothecenes degrading activity have not been yet reported.

However, the recent increasing of microorganisms isolated from trichothecenes contaminated environments and capable of transforming this class of toxins led to the assumption that this goal will not be so distant [109,110,114].

### 2.5. Zearalenone

**Figure 5:**
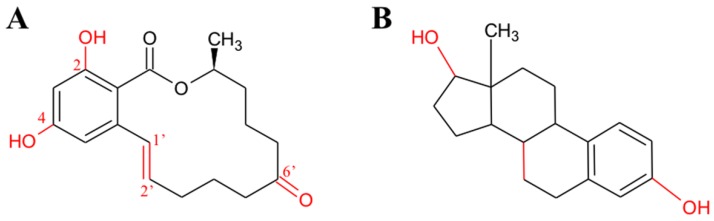
Chemical and structural analogies between zearalenone (**A**) and 17β-estradiol (**B**). The main chemical groups interacting with the estrogen receptors and responsible for zearalenone toxicity are highlighted in red (see the text for further details).

ZEN is a resorcylic acid lactone, nonsteroidal yet estrogenic mycotoxin, which binds to mammalian estrogen receptors (ER), although with lower affinity than the natural estrogens 17β-estradiol, estriol and estrone [115]. From a structural point of view, ZEN resembles the 17-βestradiol, and this similarity is responsible for its estrogenic potential and ER binding capacity (Figure 5).

In vivo ZEN undergoes reduction to α-ZEL and β-zearalenol (β-ZEL), with the first being more estrogenic than the parent compound [116]. A further reduction leads to the formation of α- and β-zearalanol (α-ZAL, β-ZAL), which are both less estrogenic than ZEN.

ZEN estrogenicity is greatly enhanced by the reduction of the 6′-ketone group and of the 1′-2′double bond, while it is reduced by the methylation of the hydroxyls in C4 and C2. The OH group in 6′of α and β-ZEL resembles that of C3 in estradiol. This group strongly interacts with ER, while the saturation of the 1′-2′double bond increases the flexibility of the molecule. This allows ZEN and/or ZEL to undergo some slight conformational changes, effective at adapting the molecules to the binding pocket of the receptor. Hydroxyl groups in C4 and C2 also contribute to the binding, with the first being more important than the latter in increasing ZEN estrogenic potential [116] (Figure 5).

ZEN and derivatives detoxification strategies aim at disrupting their estrogenic activity. Among the reported ZEN metabolizing reactions, lactone ring cleavage, catalyzed by esterases, is the prevalent detoxification method described so far. Since the resulting hydroxyketones spontaneously decarboxylate, the reaction is irreversible.

A lactonohydrolase (EC 3.1.1) from the fungus *Clonostachys rosea* has been identified as capable of degrading ZEN. The enzyme was purified to homogeneity, and its gene (namely *zhd101*) was cloned and characterized by heterologous expression in *E. coli* BL21 and *S. cerevisiae* INVSc1 [117,118] (Table 4). The recombinant protein was proved to completely degrade 2 µg/mL of ZEN in in vitro assays, although with minimal yeast mediated conversion of ZEN to β-zearalenol. *Zhd101* gene and transformants carrying lactonohydrolase gene were patented in 2002 [119].

Besides the above-mentioned activity towards AFs, laccases are also able to degrade ZEN. Novozyme patented the laccase-mediator degradation of ZEN in 2007 [120], using *Streptomyces coelicolor* LC and, preferably, phenotiazin-10-propionic acid or methylsyringate as redox mediators. By using this system, ZEN was completely removed by 0.1 mg/mL of LC and 0.2 mM of mediator at 37 °C, within 24 h. Laccase degrading capabilities were also studied by Banu and colleagues [67] using one LC-enriched *Trametes versicolor* preparation. Reactions, containing 6.2 × 10^−4^ µg/mL of ZEN, were incubated with 0.4 mg/mL of laccase for 4 h at 30 °C, achieving a maximum of 81.7% of degradation.

Another ZEN degrading oxidoreductase is a 2-Cys peroxiredoxin (Prx) (EC 1.11.1.15) extracted from *Acinetobacter* sp. SM04 [121,122]. Prxs are peroxidases containing redox-active cysteine, which can be oxidized to cystine by using H_2_O_2_ as cofactor. Prx degraded up to 95% of ZEN (20 µg/mL) in in vitro assays, when added with 20mM H_2_O_2_; nearly 90% of the toxin was instead degraded in contaminated corn sample (ZEN levels of nearly 1000 µg/mL), treated for 6 h at 30 °C with purified recombinant Prx, plus 0.09% (mass fraction, mol/mol) H_2_O_2_. Toxicity bioassays were also performed. The proliferative effect on MCF-7 cells by ZEN-DPs was reduced by 75%. Altalhi and colleagues [123] identified a 5.5 kb gene fragment from *Pseudomonas putida* pZEA-1, which encodes for DE(s) not yet characterized. *E. coli* DH5 cells, expressing the 5.5 kb gene fragment, were able to reduce 100 µg/mL of ZEN by 85% after 72 h, while no degradation was observed by wild type cells. ZEN DPs were also shown to possess a marked reduced toxicity on *Artemia salina* larvae.

## 3. Potentialities and Limitations of Mycotoxin Degrading Enzymes in Food, Feed, and Bioenergy

The use of enzymes in the food, feed, biogas and biofuel industries is not new, as biocatalysts have been increasingly used in the last 30 years. They allowed reducing the employment of hazardous chemicals, to use mild working conditions, to increase specificity, to speed up a process or to simply create new products.

Enzyme biotechnological applications are widespread: the dairy industry has a long story of protease use for cheese manufacturing, while bakery employs laccase and transglutaminase to achieve doughs strengthening; phytases are used in non-ruminant animal nutrition for the degradation of phytic acid, which interferes with mineral absorption; pretreatment of lignocellulosic biomass for bioethanol production can be achieved using a cocktail of cellulases (endoglucanase, EC 3.2.1.4, exoglucanase, EC 3.2.1.91 and cellobiohydrolase, EC 3.2.1.21) instead of employing acid or alkali treatments [124,125].

Mandatory requirements for enzyme application in large scale industry are (a) safety; (b) effectiveness; (c) low cost of production and purification for both enzyme and cofactors, if needed; d) stability to wide ranges of temperature, pH and organic solvents, and thus compatibility to productive processes. All those characteristics make the enzyme use advantageous from both technological and economic points of view. Native enzymes usually do not respond to each distinctive requirement of a perfect industrial enzyme, but these features can be achieved via molecular engineering and structure-function modifications by targeted or random mutagenesis.

The most important limitation related to the application of mycotoxin DEs in real matrices is represented by the reduced effectiveness of the process due to matrix effects. The physicochemical properties of food, such as the moisture and fat content, acidity, texture etc., greatly influence the success of the detoxification process. Moreover, inhibitory compounds may be present in raw materials and mycotoxins can occur in masked forms in plant tissues [126]; thus, their bioavailability for the enzyme catalysis may be further reduced. These implications might require pretreatments, additional time and costs, which must be taken into account in the development of industrial applications.

Despite these limitations, the potentialities of enzyme use in food and feed industries remain widespread. Their application is versatile, since they can be used both in free or immobilized form and easily applied to well established industrial processes (fermentations, ripening, brewing or cheese manufacturing, feed and bioenergy production).

Moreover, DEs can be heterologously expressed by industrial microorganisms, such as lactic acid bacteria or yeasts, to perform in situ bioremediation. New smart and edible packaging with enzymes are also under study [127].

### 3.1. Food

The European Community established the last regulation on food enzymes in 2008 [128]. This regulation covers “enzymes added to food to perform a technological function in the manufacture, processing, preparation, treatment, packaging, transport or storage of such food, including enzymes used as processing aids”. All enzymes are submitted to this regulation as processing aids, with the exception of lysozyme and invertase, considered as additives.

Article 6 states that necessary conditions to authorize a food enzyme are that (i) its use does not pose a safety concern for the health of the consumer; (ii) it responds to a technological need that cannot be achieved by other economically and technologically practicable means; and (iii) it does not mislead the consumer.

Only recently, acceptability criteria for detoxification treatments, including biotransformation, have been set for commodities intended for animal nutrition [38].

Some of the enzyme recognized to have mycotoxins degrading capabilities have been proposed and studied for applications in food industry, even if thought for other purposes.

Laccases have been studied for their introduction in bakery and dairy as crosslinking agent, in brewing, wine and fruit juice production as clarifying agent and polyphenol remover [129,130,131]. Peroxidases have also been studied for their crosslinking activity in bakery and dairy industry, but the use of H_2_O_2_ as cofactor limits their real application in food.

Amano enzyme Inc. (United Kingdom) requested EFSA to perform a scientific risk assessment on one laccase from *Trametes hirsuta*, currently in progress.

The employment of DEs in the food industry, especially in immobilized forms, is not unrealistic. Still, it must overcome the gap of knowledge related to the effects that these enzymes exert on the nutritional and organoleptic qualities of raw materials, as well as fill a legislative void, which, so far, does not foresee the possibility of detoxifying treatments for food.

### 3.2. Feed

Enzyme use in animal diets has a long history, starting from the 1920s, when a commercial enzyme preparation, produced by *Aspergillus orizae*, known as Protozyme, was applied in poultry diets [132]. Phytases, proteases, α-galactosidases, glucanases, xylanases, α-amylases, and polygalacturonases are commercially available and increasingly used in animal nutrition, with huge benefits in terms of nutritional value and micronutrients availability [133].

For authorizing their use in feed, EFSA requires solid supporting information about production, safety, efficacy and non-interference with the nutritional and organoleptic quality of the feed [134].

However, to become a commercial reality, an effective and safe DE must be formulated in order to guarantee its stability and efficacy during storage, and to ensure detoxification once released in the animal digestive tract.

There are two ways to apply a DE to feed: (a) as stabilized by a suitable formulation for becoming a feed additive or (b) used in a detoxifying process for contaminated raw materials intended for feed.

(a) During feed production enzymes are usually added before pelleting, in premixes with other additives (such as vitamins), or after pelleting as a liquid application. The most convenient approach is to introduce them as protected in formulations before pelleting: indeed, in this condition enzymes are less exposed to high moisture content and high temperature [135], thus preventing their inactivation.

High temperature protection is usually achieved by coating. Its challenging development has to succeed in both stabilizing the enzyme during feed processing, and in fast dissolving in the animal gut, thus releasing the active enzyme. To this aim new coated and tough (CT) granulated coatings have been developed by different companies (Novozymes^®^, Bagsvaerd, Denmark; DSM™, The Netherlands). Within the CT granulates, enzymes are enclosed in a core matrix of minerals and carbohydrates. The outer layer can consist of vegetable oil, kaolin, and calcium carbonate, which are readily degraded upon ingestion [136]. On 5 May 2014 Biomin^®^ (Holding GmbH) received the first-ever EFSA positive opinion, for using a purified enzyme in feed. This is an esterase, embodied in a 10% maltodextrin matrix and spray dried, capable of biotransforming fumonisins.

(b) The development of a detoxifying application at industrial scale should allow the treatment of highly contaminated batches, with significant reduction of mycotoxin under the limits imposed by the current regulation. The achieved detoxification could result worthless in case of residual contamination by toxigenic fungi, if long term storage of the treated material is expected.

Free enzymes can be delivered in the feed productive process both in liquid and in solid forms (some prior wetting in dissolving feeders might be needed), through pumps, and mixed with the raw material, usually through a vertically mounted, shaft-driven impeller. This mode of application well fits a batch type process.

The major limitation of this approach is the difficulty to achieve a homogenous enzyme delivery throughout the entire material. In addition, the enzyme must target hot spots of contamination, typical of the distribution of toxigenic fungi and associated mycotoxins.

Among the environmental parameters that should be well set to preserve enzyme catalytic properties, moisture is by far the most critical. A high water content can increase enzyme activity, but simultaneously trigger fungal spread.

Immobilized enzymes encounter the same limitations, but they present the advantage of allowing the setup of continual processes, enzyme reuse and costs reduction.

### 3.3. Bioenergy

In biofuel production, enzymes are used to break down complex carbohydrates and to release ready fermentable sugars from cheap raw materials. Cellulose hydrolysis preceding fermentation is usually performed with cellulases, cellobiohydrolases and endoglucanases [137]. Pre-treatment of lignocellulosic biomasses prior to saccharification by laccases is under study, both to degrade lignin and to detoxify phenolic inhibitory compounds in bioethanol production [138,139]. The same biomass breakdown is realized during biogas production, especially in maize-feeded biogas plants, to adapt and accommodate raw materials and to enhance their suitability for the activity of methanogenic microorganisms [140,141]. In this case, the mixture may include cellulose, hemicellulose, pectin and starch DEs, but also proteins able to breakdown long chain fatty acids, whose accumulation is responsible for the decrease of the process stability.

Despite these applications, no enzymes have been yet developed to reduce mycotoxin level in maize silage, plants or any other substrate used for bioenergy production. Their content, which mirrors fungal contamination in raw materials, affects the efficiency of the anaerobic digestion and ethanol/methane accumulation: indeed, fungal metabolites, including mycotoxins, act as antimicrobial or inhibitory compounds, or lead to excessive foam formation [142].

Storm et al. [143] recently reviewed fungal and mycotoxin contamination in maize feeding biogas plants. The authors assessed that pre-harvest contamination of grass and maize by *Fusarium* spp., *Aspergillus* spp. and *Alternaria* spp. can lead to DON, ZEN, FBs and AFs accumulation, but with concentration usually lower than regulatory limits. Salati et al. [144] reported that the addition of AFB_1_ in a lab scale anaerobic digestion trials did not affect biogas production. In a recent conference abstract presented by De Gelder et al. [145] the authors evaluated the biodegradability of mycotoxins during anaerobic digestion. They performed lab scale degradation tests with digestate spiked with different mycotoxins. After 30 days of mesophilic and thermophilic digestion, each tested mycotoxin resulted absent in the final products, suggesting the activation of some adsorption/detoxification mechanism during the fermentation. The adsorption mechanism appears the most probable, since (1) as discussed in previous paragraphs mycotoxins are greatly stable and (2) they strongly bind to soil clay minerals and organic matter present in the reactors. However, no experiment has been performed in real biogas plants under conventional operating conditions.

Recent studies demonstrated that the addition of enzymes directly into biogas reactors has no significant effect on biogas process [146], since the enzymes are quickly degraded [147]. This obviously might require a continuous addition, and thus increases costs in comparison to raw materials pre-treatment. The development of enzymatic formulates able to couple biomass breakdown and mycotoxin degrading activity could allow the simultaneous increase of biogas yield and the safe utilization of byproducts, for which mycotoxins levels are not yet regulated.

The fate of mycotoxins during biofuel production has been only recently investigated. Dzuman et al. [148], performed a study on five different batches of the ethanol–distiller’s grains and soluble (DDGS) production process. They reported a significant increase of deoxynivalenol and its glycosylated form, DON-3-glucoside, during the first part of fermentation, when hydrolytic enzymes were added. After yeast addition, total DON content rapidly decreased. They observed an opposite trend for FB_1_, since yeast addition contributed to its increase. The same results were discussed by Wu and Munkvold [149], who estimated that mycotoxins are concentrated up to three times in DDGS compared to the feeding grain.

Despite these data, the second generation of bioethanol industry is not greatly concerned by mycotoxin contamination since: (a) less susceptible commodities can be used as raw material; (b) mycotoxins occurrence in bioethanol byproducts is not yet regulated neither in Europe or in U.S. and (c) the growth and productivity of highly tolerant fermenting yeasts are only slightly influenced by mycotoxin contamination [150,151].

The re-use of contaminated biomasses for renewable energy production is raising great interest both for economic and environmental reasons. However, more detailed studies should evaluate side effects not sufficiently considered, since by-products use for animal nutrition in Europe, Canada and U.S. [152,153,154,155] is increasing.

Sosa et al. [156] performed a preliminary economic analysis evaluating the feasibility of using fumonisin highly contaminated corns as feedstock for ethanol production. The authors calculated that the advantages deriving from the lower price of contaminated raw materials is balanced by the increase of the operating costs and the decrease of the final ethanol production by the fermenter. Thus, the performance of the process must be optimized, using process alternatives (e.g., both microfiltration and pervaporation membranes) coupled by the introduction of ethanol tolerant yeast strains, in order to effectively increase the economic value of the process.

DDGS are considered valuable by-products, thanks to the low carbon-nitrogen ratio and their high proteins and micronutrients content [157]. However, their utilization should be responsibly addressed, since ecological outcomes of mycotoxin contaminated fertilizers and health effects are still poorly investigated.

As for biogas production, the implementation of DEs for biofuel application has not been developed. Still, it benefits of less limitations than those encountered by the food and feed industries. Cloning DEs in industrial hosts could ideally allow to simultaneously perform detoxification and fermentation of contaminated biomasses.

## 4. Conclusions and Future Perspectives

Within this review we gave a global view of the identified mycotoxins DEs, their mechanisms, their current and possible application in food, feed, biogas and biofuel industries. We also presented patented commercial preparations used by feed industries.

Although this paper is focused on the main mycotoxins, for which a high level of contamination and health implications have driven the majority of economic and research efforts, the discovery of novel DEs is becoming an interesting and stimulating topic also in relation to other mycotoxins.

Several patents have been deposited, mostly in U.S. and China, describing microorganisms and methods to degrade minor mycotoxins, such as patulin [158], beauvericin [159] and moniliformin [160] in plants, maize or grains. These patents are mainly related to microorganisms or crude extracts preparation, and no enzymes have been yet characterized as responsible for the degrading activity.

It should not be excluded that enzymes efficient towards minor mycotoxins could be effective towards structurally similar main ones.

In addition to the previously described ones, another challenge related to the application of DEs, is represented by mycotoxins co-occurrence, which should require the simultaneous use, and thus optimization, of different preparations and protocols. To overcome this barrier, the combination of different strategies, such as adsorbents mixture, binding microorganisms and enzymes, suitable for the inclusion within the same procedure, should be considered.

In the optimization process, great potentialities are ascribed to structural modelling and design of experiments (DOE) technologies. These approaches are able to identify structural determinants responsible for the degradation mechanism and to improve substrate-enzyme affinity by setting the conditions, which maximize enzyme efficiency. In combination with targeted mutagenesis methods, they strongly reduce lab-optimization processes and accelerate the industrial scale up.

Transcriptional analysis of degrading microorganisms [161] has recently led to the identification of a DE from the novel *Acinetobacter* sp. *neg-1* species, described by Fanelli et al. [162] as capable of degrading OTA. This high-throughput approach, as other advanced Next Generation Sequencing technologies, such as functional metagenomic, could give a huge contribution in the identification of similar classes of DEs from complex and unexplored environments, such as contaminated soils, water or rumen. The advantage of these approaches is represented by the possibility to investigate non-culturable organisms, as well as those living in complex environmental niches, hardly analyzable under laboratory conditions.

The application of DEs could be simplified by the biotechnological advances in the field of immobilization and encapsulation techniques. Finally, an interesting perspective is represented by the development of transgenic crops able to counteract mycotoxins formation in the field. EU does not authorize the genetic modification of plants as a breeding technique. In contrast, the U.S. have 20 years of history of genetically engineered crops: herbicide-tolerant and insect-resistant crops were introduced in 1996 and today more than 90% of the corn cultivated in the U.S. is genetically modified [163].

Syngenta patented trichothecene-resistant transgenic plants bearing the *Fusarium graminearum* Tri101 gene [164], which encodes for a 3-*O*-acetyltransferase [165,166], which catalyzes the transfer of an acetate to the C3 position of trichothecenes. The company performed field trials, in Canada, the U.S., Argentina, and three European countries [108], proving that the development of self-defending crops is already achievable.

## Figures and Tables

**Table 1:** Aflatoxins (AFs) degrading enzymes.

Enzyme	Accession/EC	Producing Organism	AF Target	Toxin Concentration	In Vitro/*In Matrix* Degrading Conditions	In Vitro/*In Matrix* Degradation	Toxicity/Mutagenicity Test	Reference
aflatoxin oxidase enzyme (AFO)	EC 1.1	*Armillariella tabescens*	AFB_1_	0.05 µg/mL	■ PBS buffer 0.02 M pH 6; ■ incubation at 28 °C for 30 min	■ 100% with 0.2 mg of enriched preparation; ■ NQ* with pure enzyme	■ reduced liver toxicity in rats; ■ reduced mutagenicity in *Salmonella typhimurium* TA 98; ■ reduced genotoxicity on chicken embryos	[51]
peroxidase	EC 1.11.1.7	horseradish *(Armoracia rusticana)*	AFB_1_	312 µg/mL	■ phosphate buffer 50 mM pH 6; ■ incubation at 20 °C for 60 min; ■ 0.2U/mL of enzyme	42.2%	reduced toxicity on *Bacillus megaterium*	[52]
				440 µg/mL	■ 100 g of defatted groundnut kernels; ■ 2-16U of enzyme; ■ 50 mM phosphate buffer pH 6 and 20 mM hydrogen peroxide; ■ incubation at room temperature up to 24 h	41.1%		
laccase	EC 1.10.3.2	*Trametes versicolor* (commercial enzyme from Sigma-Aldrich, Missouri, U.S.)	AFB_1_	1.40 µg/mL	■ phosphate buffer 0.2 M pH 6.5; ■ 1 U/mL of enzyme ■ incubation at 30 °C for 72 h	87%	reduced mutagenicity on *Salmonella typhimurium* TA 100	[53]
laccase	EC 1.10.3.2	*Streptomyces coelicor*	AFB_1_	9.36 µg/mL	■ sodium acetate buffer 100 mM pH 7; ■ 0.1 mg/mL of enzyme protein and 0.2 mM mediator; ■ incubation at 37 °C for 24 h	100%	n.p.	[54]
F420H2-dependent reductases	E.C. 1.5.8	*Mycobacterium smegmatis*	AFB_1_ AFB_2_ AFG_1_ AFG_2_	n.p.	n.p.	n.p.	n.p.	[55]
Mn peroxidase	EC 1.11.1.7	*Pleurotus ostreatus*	AFB_1_	0.31 µg/mL	■ sodium lactate buffer 50 mM pH 4.5; ■ 1.5 U/mL of enzyme ■ incubation at 30 °C for 48 h	90%	n.p.	[56]
aflatoxin degradation enzyme	n.p.	*Pleurotus ostreatus*	AFB_1_	5 µg/mL	■ sodium acetate buffer 0.1 M pH 5; ■ incubation at 25 °C for 1 h	n.q.	n.p.	[50]
myxobacteria aflatoxin degrading enzyme (MADE)	n.p.	*Myxococcus fulvus* ANSM068	AFB_1_	0.1 µg/mL	■ citrate phosphate buffer 0.1 M pH 6; ■ 100 U/mL of enzyme ■ incubation at 30 °C for 48 h	72% with culture filtrates	n.p.	[57]
			AFG_1_			97%		
			AFM_1_			96%		
laccase (lac2)	EC 1.10.3.2	*Pleurotus pulmonarius* (ITEM 17144)	AFB_1_	1 µg/mL	■ sodium acetate buffer 1mM pH 5; ■ 5 U/mL enzyme and redox mediator; ■ incubation at 25 °C for 72 h	90%	n.p.	[58]
			AFM_1_	0.05 µg/mL		100%		
Ery4	CAO79915.1/EC 1.10.3.2	*Pleurotus eryngii* (PS419)	AFM_1_	0.05 µg/mL	■ sodium acetate buffer 1 mM pH 5; ■ 5 U/mL enzyme and redox mediator; ■ incubation at 25 °C for 72 h	100%	n.p.	[59]
					■ artificially spiked skim UHT milk; ■ 5 U/mL enzyme and redox mediator; ■ incubation at 25 °C for 72 h			

n.q. = not quantitative; n.p.= not provided.

**Table 2:** Fumonisin B_1_ degrading enzymes.

Enzyme	Producing Organism	Accession/EC	Toxin Concentration	Degrading Conditions	Degradation	Reference
carboxylesterase and aminotransferase	*Sphingomonas* sp. ATCC55552	E.C. 3.1.1, E.C. 2.6.1	1000 µg/mL	■ citrate buffer 0.1 M pH 3 or citrate-phosphate buffer 0.1 M pH 4; ■ overnight incubation at 37 °C	100%	[89]
carboxylesterase B and aminotransferase	*Sphingopyxis* sp. MTA144	E.C. 3.1.1, E.C. 2.6.1/ FJ426269.1	3.6 µg/mL	■ Tris-HCl 20 mM pH 8, 0.1 mg/mL BSA; ■ incubation at 30 °C for 2 h	100%	[80]
fumonisin esterase	*Sphingopyxis* sp. MTA144	E.C. 3.1.1.87	60 µg/mL	■ unspecified buffer pH 8; ■ 18 U/L of enzyme ■ incubation at 30 °C for 15 min	100% conversion to HFB_1_	[91]

**Table 3:** Trichothecenes degrading enzymes.

Enzyme	Accession/EC	Producing Organism	Trichothecene Target	Toxin Concentration	Degrading Conditions	In Vitro Degradation	Toxicity-Mutagenicity Test	Reference
cytochrome P450 system (Ddna + Kdx + KdR)	E.C. 1.14 AB744215.1 AB744217.1	S*phingomonas* sp. strain KSM1	DON	99.86 µg/mL	■ potassium phosphate buffer 10 mM pH 7.5; ■ 10% glycerol, 0.2 μM DdnA, 1.2 μM Kdx, 1.2 μM KdR, 1 mM NADH, and 100 mg/ml bovine liver catalase; ■ overnight incubation at 30 °C	100% after 3 days	reduced phytotoxicity to wheat	[112]
			NIV	105.25 µg/mL		100% after 5 days		
UDP-glycosyltransferase	AC006282	*Arabidopsis thaliana*	DON	n.p.	n.p.	n.p.	increased resistance in transgenic *Arabidopsis*	[113]

n.p. = not provided.

**Table 4:** Zearalenone degrading enzymes.

Enzyme	Producing Organism	EC	Toxin Concentration	Degrading Conditions	Degradation	Toxicity-Mutagenicity Test	Reference
laccase	*Trametes versicolor* (commercial enzyme from Sigma-Aldrich)	EC 1.10.3.2	6.2 × 10^−4^ µg/mL	■ sodium acetate buffer 0.2 M pH 5.2; ■ incubation at 30 °C for 4 h.	up to 58 %	n.p.	[67]
laccase	*Streptomyces coelicolor*	EC 1.10.3.2	9.36 µg/mL	■ sodium acetate 0.1 M pH 4.5; ■ 0.2 mM mediator; ■ incubation at 37 °C for 24 h	100%	n.p.	[120]
lactono hydrolase	*Clonostachys rosea*	E.C. 3.1.1	2 µg/mL	■ YPD; ■ incubation at 28 °C for 4 h or at 37 °C for 2 h	100%	n.p.	[117,118]
2cys-peroxiredoxin	*Acinetobacter* sp. SM04	EC 1.11.1.15	20 µg/mL	■ Tris-HCl 50 mM pH 9; ■ H_2_O_2_ ≥ 20 mM; ■ incubation at 30 °C for 4 h	up to 95%	reduced MCF-7 cells proliferation by 75%	[121]
			1 µg/mL	■ 1 mL of 0.8 M H_2_O_2_ and 19 mL of purified recombinant Prx solution pH 9; ■ incubation at 40 °C for 6 h.	up to 90%		

## Abbreviations

^1^H-NMR	Proton Nuclear Magnetic Resonance
AFB_1_	aflatoxin B_1_
AFD_1_	aflatoxin D_1_
AFL	aflatoxicol
AFM_1_	aflatoxin M_1_
AFO	aflatoxin oxidase enzyme
AFQ_1_	aflatoxin Q_1_
AFs	aflatoxins
AP1	aminopentol 1
CPA	carboxypeptidase A
CPY	carboxypeptidase Y
CT	coated and tough
DDGS	distiller’s grains and soluble
DE	degrading enzyme
DOE	Design of experiments
DON	deoxynivalenol
DPs	degradation products
EDC	endocrine disrupting chemical
EFSA	European Food Safety Authority
ER	estrogen receptors
FB_1_	fumonisin B_1_
GAPs	Good Agricultural Practices
HFB	hydrolyzed FB_1_
HR-ESI-MS	High Resolution electrospray ionization mass spectrometry
HT-2	HT-2 toxin
IARC	International Agency of Research on Cancer
LCs	laccases
MADE	Myxobacteria Aflatoxin Degrading Enzyme
MnPs	Manganese Peroxidases
NIV	nivalenol
OTA	ochratoxin A
OTα	ochratoxin α
Phe	phenylalanine molecule
Prx	peroxiredoxin
T-2	T-2 toxin
ZEN	zearalenone
α-ZAL	α-zearalanol
α-ZEL	α-zearalenol
β-ZAL	β-zearalanol
β-ZEL	β-zearalenol
